# Vitamin D Trajectories and Cardiometabolic Risk Factors During Childhood: A Large Population-Based Prospective Cohort Study

**DOI:** 10.3389/fcvm.2022.836376

**Published:** 2022-03-16

**Authors:** Pei Xiao, Hong Cheng, Haibo Li, Xiaoyuan Zhao, Dongqing Hou, Xianghui Xie, Jie Mi

**Affiliations:** ^1^Center for Non-communicable Disease Management, National Center for Children’s Health, Beijing Children’s Hospital, Capital Medical University, Beijing, China; ^2^Department of Epidemiology, Capital Institute of Pediatrics, Beijing, China; ^3^Division of Birth Cohort Study, Fujian Maternity and Child Health Hospital, Affiliated Hospital of Fujian Medical University, Fuzhou, China

**Keywords:** vitamin D, childhood, cardiometabolic risk factors, cohort study, dyslipidemia

## Abstract

**Background and Objectives:**

Vitamin D has been indicated to play an important role in the optimal function of the cardiovascular system. However, with limited evidence, it remains unclear whether vitamin D status transition during childhood would affect cardiometabolic risk factors. Thus, we aimed to identify the associations of the longitudinal trajectory of vitamin D status with cardiometabolic risk factors in children.

**Methods:**

A total of 10,482 participants with complete follow-up records from a large population-based prospective cohort study were included in this analysis. The 25-hydroxyvitamin D [25(OH)D] concentrations, blood pressure, blood lipids, and fasting blood glucose were determined. Vitamin D deficiency was defined as serum 25(OH)D concentrations below 30 nmol/L according to the Institute of Medicine recommendations. Based on the vitamin D status at baseline and follow-up, we identified four possible trajectories: (1) persistent non-deficiency (reference); (2) baseline non-deficiency to follow-up deficiency; (3) baseline deficiency to follow-up non-deficiency; (4) persistent deficiency. The relationships between cardiometabolic risk factors and vitamin D trajectories were evaluated using adjusted risk ratios (*RRs*).

**Results:**

Overall, 35.1 and 24.2% of participants had vitamin D deficiency at the baseline and follow-up, respectively, and 15.1% were under the condition of persistent vitamin D deficiency. Compared to children with persistent non-deficiency, those who shifted from non-deficiency at baseline to deficiency at follow-up had a 2.09-fold (95% *CI*: 1.36, 3.23) increased risk of high triglyceride (TG). Besides, children with altered vitamin D status from deficiency to non-deficiency during follow-up were still at a significantly higher risk of high total cholesterol (TC) than the reference group [*RR* (95% *CI*): 1.39 (1.04, 1.86)]. Finally, children with persistent vitamin D deficiency were at the highest risks of high TC [*RR* (95% *CI*): 1.61 (1.18, 2.19), *P_*trend*_* < 0.001], high low-density lipoprotein cholesterol (LDL-C) [*RR* (95% *CI*): 1.53 (1.04, 2.27), *P_*trend*_* = 0.046], and high TG [*RR* (95% *CI*): 1.96 (1.34, 2.87), *P_*trend*_* = 0.003].

**Conclusion:**

Our results suggest that persistent vitamin D deficiency might increase the risk of dyslipidemia in children, and vitamin D deficiency could have has short- and long-term effects on TG and TC, respectively.

## Introduction

The development of cardiovascular disease begins with cardiometabolic risk factors such as hypertension, hyperglycemia, and dyslipidemia. More importantly, cardiometabolic risk factors occurring in childhood significantly increase the risk of cardiovascular events in adulthood (1). Our previous cohort study showed that, with a follow-up period of over 2 years, the incidence rates of hypertension, hyperglycemia, and dyslipidemia across weight status groups in Chinese children aged 6–17 years had reached 7.0–26.3%, 7.6–11.2%, and 7.2–17.9%, respectively (2). It is well demonstrated that nutrition is linked to aging and cardiovascular health in humans (3). Vitamin D, an essential nutrient for calcium and phosphate homeostasis, has been shown to play an important role in the optimal function of the cardiovascular system (4). However, vitamin D deficiency is currently high prevalence worldwide (5), particularly in children (6). Thus, it has become a major public health issue that poses a serious threat to children’s health.

The associations between vitamin D deficiency and cardiometabolic risk factors in children have been reported in previous studies (7–9). Results from the National Health and Nutrition Examination Survey showed that low high-density lipoprotein cholesterol (HDL-C) and hyperglycemia were associated with vitamin D deficiency in United States children (8). In another cross-sectional study from China, vitamin D deficiency was revealed to be correlated with high triglyceride (TG) and have a synergistic effect with obesity on hyperglycemia (7). However, the potential effects of the vitamin D status transition in childhood on cardiometabolic risk factors remain unclear. Evidence derived from prospective studies is still relatively scarce to support causal links between vitamin D and cardiometabolic risk factors in children.

Therefore, using a large population-based prospective cohort, we aimed to identify the associations between the longitudinal trajectory of vitamin D status and cardiometabolic risk factors (including hypertension, hyperglycemia, and dyslipidemia) in children. We also looked into the associations of persistent vitamin D deficiency status with cardiometabolic risk factors and the modification of persistent obesity on these associations.

## Materials and Methods

### Study Population

**FIGURE 1:**
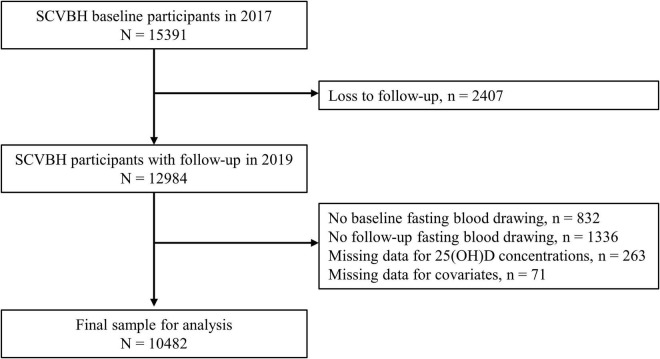
Flow diagram of SCVBH cohort and analytic sample.

Data were obtained from a population-based prospective cohort study: The School-based Cardiovascular and Bone Health Promotion Program (SCVBH). The original aim of SCVBH was to explore the risk factors of cardiovascular and bone health in school-age children by dynamically collecting life behavior, nutrition, and body composition information. A detailed description of the protocol and baseline survey results has been reported elsewhere (10). Briefly, a stratified cluster sampling method was used to select students in 30 schools (8 primary schools, 21 high schools, and a 12-year education school) from four districts of Beijing, including Dongcheng, Tongzhou, Fangshan, and Miyun. All the students in Grades 1–4 of primary schools and Grade 1 of junior and senior high schools were invited to participate in the baseline survey from November to December in 2017. 15,391 students aged 6–16 were enrolled in the questionnaire survey and physical examination at baseline, and 14,396 of them agreed to have a blood sample collection. The follow-up investigation with the same contents of baseline was conducted in the cohort from November to December in 2019, and 12,984 participants were successfully followed up with a follow-up rate of 84%. After excluding participants without blood draw in either of the two surveys and those who had missing data of analytical variables, a total of 10,482 participants were included in this analysis. The flowchart of the excluded participants along with the corresponding reason is summarized in Figure 1.

The study protocol was approved by the Institutional Review Boards of Capital Institute of Pediatrics (approval number: SHERLL2016026), and written informed consent was obtained from the parents of participants.

### Data Collection

A self-administered questionnaire, which had been pretested in a pilot study (11), was used to collect personal information, including socio-demographic characteristics (e.g., sex, age, and grade), lifestyle factors (e.g., smoking, drinking, physical activity, and dietary habits), and personal/family medical history. Subjects answered the questions mainly by themselves or assisted by their parents/guardians when necessary (for those under 10 years old). Information on smoking and drinking behavior was collected by asking whether the participants had drunk ≥ a standard amount of alcoholic beverage (e.g., 120 ml wine, 50 ml liquor, or a bottle of beer) and smoked at least one complete cigarette in the past month. According to the cardiovascular health promotion statement in children from the American Heart Association (12), we defined smoking status as having smoked more than one complete cigarette in the past month. Drinking referred to the behavior of ≥ a standard amount of alcoholic beverage consumption in the past month based on the Youth Risk Behavior Surveillance System method (13). Physical activity was assessed by the questions of the frequency (per week) and duration (minutes) of vigorous or moderate activity. Ideal physical activity was defined as a daily frequency of moderate or vigorous physical activity ≥60 min (12). A structured dietary survey component was used to collect the frequency of different food intakes in the past half-year. Ideal dietary behaviors were defined as achieving at least 4 out of 5 following items: (1) fruits and vegetables (≥1 time/day); (2) aquatic foods (≥1 time/week); (3) whole-grain foods (≥1 time/day); (4) sugar-sweetened beverage (<1 time/week); and (5) bean-curd or dairy products (≥1 time/day) (12). Children whose parents ever had hypertension, diabetes, or dyslipidemia were identified as having a family history of cardiometabolic risk factors. The usage of vitamin D supplementation was assessed by asking whether the subjects had taken vitamin D supplements in the past half-year. Sexual maturity was determined by the occurrence of spermatorrhea in boys and menstruation in girls.

Standing height and weight without shoes were measured to 0.1 cm by the stadiometer and 0.1 kg by the electronic scale, respectively. Body mass index (BMI) was calculated as weight (kg) divided by the square of height (m^2^). Resting blood pressure (BP) was measured from the right brachial artery three times with 1–2 min intervals (OMRON HBP-1300, Omron, Kyoto, Japan) in a sitting position, and the average of the last two readings was used for analysis.

### Laboratory Assay

Blood samples were drawn after an overnight fast and centrifuged for 10 min at 1509.3 × *g*. Serum specimens were collected and then stored at -80°C in the central clinical laboratory until analysis. The serum 25-hydroxyvitamin D [25(OH)D] concentrations were measured by chemiluminescent immunoassay (DiaSorin, Stillwater, MN, United States). Fasting glucose (enzyme hexokinase method) and blood lipids (enzymatic methods) were measured using the Hitachi 7080 biochemistry autoanalyzer (Hitachi, Tokyo, Japan).

### Variables Definition

Hypertension was defined as systolic blood pressure (SBP) and/or diastolic blood pressure (DBP) ≥ 95th sex-, age- and height-specific percentiles (Supplementary Tables 1, 2) for Chinese children and adolescents, or taking anti-hypertensive drugs (14). Dyslipidemia, including high total cholesterol (TC), high low-density lipoprotein cholesterol (LDL-C), high TG, and low HDL-C, was diagnosed by the sex- and age-specific lipoprotein cutoff points (Supplementary Table 3) for Chinese children and adolescents (15), or taking anti-hyperlipidemic medications. Fasting blood glucose ≥5.6 mmol/L (16), or taking anti-hyperglycemic medications was regarded as hyperglycemia. Obesity was diagnosed by the gender- and age-specific BMI cutoff points proposed by the International Obesity Task Force (17). Vitamin D deficiency was defined as serum levels of 25(OH)D below 30 nmol/L according to the Institute of Medicine recommendations (18).

Persistent cardiometabolic risk factors, obesity, and vitamin D deficiency were defined as having the corresponding status at baseline and follow-up. We identified four possible vitamin D trajectories: (1) persistent non-deficiency (reference): baseline 25(OH)D ≥ 30 nmol/L and follow-up 25(OH)D ≥ 30 nmol/L; (2) non-deficiency to deficiency: baseline 25(OH)D ≥ 30 nmol/L and follow-up 25(OH)D < 30 nmol/L; (3) deficiency to non-deficiency: baseline 25(OH)D < 30 nmol/L and follow-up 25(OH)D ≥ 30 nmol/L; (4) persistent deficiency: baseline 25(OH)D < 30 nmol/L and follow-up 25(OH)D < 30 nmol/L (18).

### Statistical Analysis

Categorical variables were presented as frequency (%), and continuous variables were expressed as mean (*SD*) or median (*IQR*) based on their distributions. Differences in characteristics across vitamin D status groups were examined by Student’s *t*-test (normally distributed data), the Mann-Whitney *U* test (skewed data), and Pearson χ*^2^* test (categorical data). The cumulative incidence rates (*CIRs*) for cardiometabolic risk factors were calculated as the number of new cases within a 2-year follow-up divided by the number of participants without corresponding risk factors at baseline.

Sex-specific generalized additive models with smoothing spline terms were applied to explore the non-linear associations between the changes of 25(OH)D concentrations during 2-year follow-up (by subtracting its concentrations at baseline from those at follow-up) and cardiometabolic risk factors. The relationships between cardiometabolic risk factors and vitamin D trajectories were determined by risk ratios (*RRs*) derived from multivariable log-binomial regressions. The trends of risks across groups were examined by treating the trajectories as continuous variables. We further divided the participants into four groups based on whether they had persistent vitamin D deficiency and persistent obesity to evaluate their joint effects on cardiometabolic risk factors, the interactions between them were assessed by adding a two-way product term to the models. Odds ratios (*ORs*) derived from multivariable logistic regression were used to assess the associations between persistent cardiometabolic risk factors and persistent vitamin D deficiency. In the multivariable adjustments mentioned above, sex (except for sex-stratified analysis), age, smoking, drinking, diet habitats, physical activity, family history of cardiometabolic risk factors, the usage of vitamin D supplements, sexual maturity status, and baseline cardiometabolic parameters were treated as covariates. The baseline cardiometabolic parameters were transformed into sex- and age-specific *Z*-scores to eliminate their variability with growth and development.

A two-tailed *P* ≤ 0.05 was considered statistically significant, and all analyses were performed by *R* software (version 3.4.0)^1^.

## Results

### Characteristics of the Study Population

A total of 10,482 children (5,202 boys, 49.6%) with a mean age of 10.9 (±3.3) years were included in the analysis. Overall, 35.1 and 24.2% of them had vitamin D deficiency at baseline and follow-up, respectively, and 15.1% were with a persistent vitamin D deficiency condition. The characteristics stratified by baseline vitamin D status are presented in Table 1. Children with vitamin D deficiency at baseline were in a worse condition of cardiometabolic parameters at follow-up (except for SBP and FBG), for example, having higher TC (4.06 vs. 4.00 mmol/L), LDL-C (2.29 vs. 2.24 mmol/L), and lower HDL-C (1.41 vs. 1.43 mmol/L) when compared to vitamin D non-deficiency children. Besides, they were less likely to use vitamin D supplements and achieve the ideal physical activity and dietary behaviors.

**TABLE 1:** Characteristics of study population according to vitamin D status at baseline.

	Vitamin D status at baseline			
Characteristics	Deficiency (<30 nmol/L)	Non-deficiency (≥30 nmol/L)	*P-*value^a^	All
No. (%)	3684 (35.1)	6798 (64.9)		10482 (100.0)
Age, mean (SD), year	11.6 (3.2)	10.5 (3.3)	<0.001	10.9 (3.3)
Girls, no. (%)	2191 (59.5)	3089 (45.4)	<0.001	5280 (50.4)
Smoking, no. (%)	39 (1.1)	82 (1.2)	0.562	121 (1.2)
Drinking, no. (%)	292 (7.9)	403 (5.9)	<0.001	695 (6.6)
Ideal physical activity, no. (%)	177 (4.8)	393 (5.8)	0.039	570 (5.4)
Ideal dietary behaviors, no. (%)	618 (16.8)	1358 (20.0)	<0.001	1976 (18.9)
Family history of cardiometabolic risk factors, no. (%)	1187 (32.2)	2023 (29.8)	0.01	3210 (30.6)
Usage of vitamin D supplements, no. (%)	577 (15.7)	1503 (22.1)	<0.001	2080 (19.8)
Sexual maturity, no. (%)	1836 (49.8)	2244 (33.0)	<0.001	4080 (38.9)
Parameters in baseline				
25(OH)D, mean (SD), nmol/L	24.0 (4.2)	41.9 (10.1)	<0.001	35.6 (12.0)
BMI, mean (SD), kg/m^2^	20.7 (4.8)	20.0 (4.6)	<0.001	20.2 (4.6)
SBP, mean (SD), mmHg	110.4 (11.5)	109.8 (11.3)	0.005	110.0 (11.4)
DBP, mean (SD), mmHg	60.6 (7.4)	59.9 (7.3)	<0.001	60.2 (7.3)
FBG, mean (SD), mmol/L	5.17 (0.48)	5.14 (0.38)	<0.001	5.15 (0.41)
TC, mean (SD), mmol/L	3.93 (0.74)	3.90 (0.74)	0.078	3.91 (0.74)
LDL-C, mean (SD), mmol/L	2.23 (0.61)	2.18 (0.60)	<0.001	2.19 (0.61)
HDL-C, mean (SD), mmol/L	1.37 (0.32)	1.41 (0.32)	<0.001	1.40 (0.32)
TG, median (IQR), mmol/L	0.77 (0.56, 1.05)	0.69 (0.50, 0.93)	<0.001	0.72 (0.52, 0.97)
**Parameters at follow-up**				
25(OH)D, mean (SD), nmol/L	33.6 (12.2)	45.5 (15.0)	<0.001	41.3 (15.2)
Vitamin D deficiency, no. (%)	1582 (14.0)	952 (42.9)	<0.001	2534 (24.1)
BMI, mean (SD), kg/m^2^	21.8 (5.1)	21.1 (4.8)	<0.001	21.3 (4.9)
SBP, mean (SD), mmHg	112.8 (11.7)	112.4 (12.2)	0.167	112.5 (12.0)
DBP, mean (SD), mmHg	62.7 (7.7)	61.8 (7.5)	<0.001	62.1 (7.6)
FBG, mean (SD), mmol/L	5.12 (0.45)	5.15 (0.40)	0.001	5.14 (0.42)
TC, mean (SD), mmol/L	4.06 (0.75)	4.00 (0.74)	<0.001	4.02 (0.75)
LDL-C, mean (SD), mmol/L	2.29 (0.61)	2.24 (0.60)	<0.001	2.26 (0.60)
HDL-C, mean (SD), mmol/L	1.41 (0.27)	1.43 (0.29)	<0.001	1.42 (0.28)
TG, median (IQR), mmol/L	0.67 (0.49, 0.96)	0.62 (0.46, 0.88)	<0.001	0.64 (0.47, 0.91)

*BMI, body mass index; SBP, systolic blood pressure; DBP, diastolic blood pressure; FBG, fasting blood glucose; TC, total cholesterol; LDL-C, low-density lipoprotein cholesterol; HDL-C, high-density lipoprotein cholesterol; TG, triglycerides; SD, standard deviation; IQR, interquartile range.*

*^a^P-value represents differences in means ± SD or proportions using Student’s t-test, the Mann–Whitney U test or Pearson χ^2^ test.*

### Associations Between Vitamin D Trajectories and Cardiometabolic Risk Factors

**FIGURE 2:**
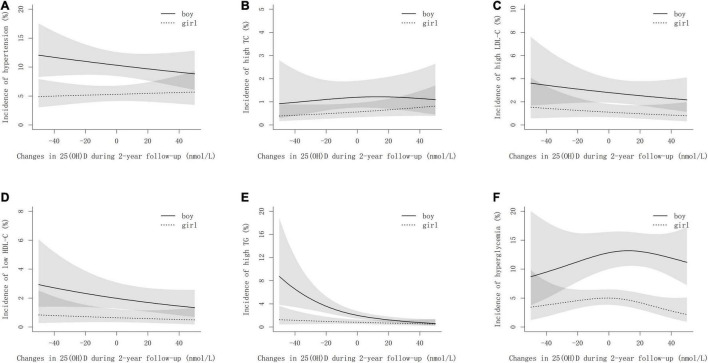
Non-linear associations between the incidences of cardiometabolic risk factors and changes in 25(OH)D during follow-up by sexes. **(A–F)** stand for hypertension, high TC, high LDL-C, low HDL-C, high TC, and hyperglycemia, respectively. The models are adjusted for age, smoking, drinking, physical activity, dietary habits, BMI, family history of cardiometabolic risk factors, vitamin D supplements, sexual maturity, and baseline cardiometabolic parameters. Changes in 25(OH)D are determined by 25(OH)D concentrations at follow-up minus that at baseline.

Figure 2 shows the sex-specific non-linear associations of changes in 25(OH)D concentrations during the 2-year follow-up with the risk of cardiometabolic risk factors after multivariate adjustment. The risk of developing high TG decreased exponentially with the increase of 25(OH)D during follow-up, particularly in boys (Figure 2E). For high LDL-C and low HDL-C, the risks decreased linearly as 25(OH)D increased (Figures 2C,D). However, regarding hypertension, a negative linear association with the increment of 25(OH)D within the follow-up period was observed in boys only (Figure 2A). Interestingly, an inverted U-shaped relationship was noticed between hyperglycemia and increased 25(OH)D (Figure 2F).

The adjusted *RRs* for cardiometabolic risk factors across different vitamin D trajectories were calculated using log-binomial regression analyses (Table 2). Children with persistent vitamin D deficiency were at the highest risks of high TC [*RR* (95% *CI*): 1.61 (1.18, 2.19), *P_*trend*_* < 0.001], high LDL-C [*RR* (95% *CI*): 1.53 (1.04, 2.27), *P_*trend*_* = 0.046], and high TG [*RR* (95% *CI*): 1.96 (1.34, 2.87), *P_*trend*_* = 0.003]. Children who shifted vitamin D status from non-deficiency at baseline to deficiency at follow-up had a 2.09-fold increased risk (95% *CI*: 1.36, 3.23) of high TG than those with persistent non-deficiency (reference group). However, children who altered vitamin D status from deficiency to non-deficiency during follow-up were still at a significantly higher risk of high TC than the reference group [*RR* (95% *CI*): 1.39 (1.04, 1.86)].

**TABLE 2:** Multivariate adjusted risk ratios (95% *CI*)^a^ of cardiometabolic abnormities according to the longitudinal trajectory of vitamin D status from baseline to follow-up.

	Vitamin D deficiency^b^						
Cardiometabolic risk factors	Baseline	Follow-up	Case	*N*	*CIR* (95% *CI*)	*RR* (95% *CI*)	*P*_*trend*_ ^c^
**Hypertension**							0.268
	–	–	557	4,871	11.4 (10.6∼12.4)	1.00 (Ref)	
	–	+	79	839	9.4 (7.5∼11.6)	0.96 (0.75∼1.24)	
	+	–	190	1,777	10.7 (9.3∼12.2)	0.94 (0.80∼1.11)	
	+	+	129	1,370	9.4 (7.9∼11.1)	0.90 (0.73∼1.11)	
**Hyperglycemia**							0.527
	–	–	460	5,272	8.7 (8.0∼9.5)	1.00 (Ref)	
	–	+	61	820	7.4 (5.7∼9.5)	0.94 (0.71∼1.25)	
	+	–	156	1,877	8.3 (7.1∼9.7)	0.99 (0.82∼1.19)	
	+	+	119	1,383	8.6 (7.2∼10.2)	1.11 (0.89∼1.39)	
**High TC**							<0.001
	–	–	131	5,717	2.3 (1.9∼2.7)	1.00 (Ref)	
	–	+	32	928	3.4 (2.4∼4.8)	1.04 (0.70∼1.56)	
	+	–	72	2,036	3.5 (2.8∼4.4)	1.39 (1.04∼1.86)*	
	+	+	76	1,530	5.0 (3.9∼6.2)	1.61 (1.18∼2.19)**	
**High LDL-C**							0.046
	–	–	144	5,710	2.5 (2.1∼2.9)	1.00 (Ref)	
	–	+	14	926	1.5 (0.8∼2.5)	0.96 (0.54∼1.69)	
	+	–	56	2,034	2.8 (2.1∼3.6)	1.15 (0.84∼1.57)	
	+	+	38	1,532	2.5 (1.8∼3.4)	1.53 (1.04∼2.27)*	
**Low HDL-C**							0.056
	–	–	171	4,995	3.4 (2.9∼4.0)	1.00 (Ref)	
	–	+	44	838	5.3 (3.8∼7.0)	1.33 (0.93∼1.89)	
	+	–	56	1,770	3.2 (2.4∼4.1)	0.91 (0.67∼1.24)	
	+	+	37	1,376	2.7 (1.9∼3.7)	0.68 (0.47∼1.01)	
**High TG**							0.003
	–	–	130	5,656	2.3 (1.9∼2.7)	1.00 (Ref)	
	–	+	29	917	3.2 (2.1∼4.5)	2.09 (1.36∼3.23)***	
	+	–	51	1,986	2.6 (1.9∼3.4)	1.23 (0.89∼1.71)	
	+	+	43	1,502	2.8 (2.1∼3.8)	1.96 (1.34∼2.87)***	

*CIR, cumulative incidence rate; RR, risk ratio; CI, confidence interval; TC, total cholesterol; LDL-C, low-density lipoprotein cholesterol; HDL-C, high-density lipoprotein cholesterol; TG, triglycerides.*

*^a^Model was adjusted for age, sex, smoking, drinking, physical activity, dietary habits, BMI, family history of cardiometabolic risk factors, vitamin D supplements, and sexual maturity.*

*^b^Low vitamin D was defined as 25(OH)D < 30 nmol/L.*

*^c^P_trend_ was calculated by treating the trajectories as a continuous variable in the log-binomial model.*

*0.01 ≤ P < 0.05; **0.001 ≤ P < 0.01; ***P < 0.001.

### Associations of Cardiometabolic Risk Factors With Persistent Vitamin D Deficiency and Persistent Obesity

The joint effects of persistent vitamin D deficiency and persistent obesity on cardiometabolic risk factors are shown in Table 3, and no multiplicative interaction between them (all *P*_*interaction*_ > 0.05) was observed. However, among the children who were persistently non-obese during the whole investigation period, the risk of high TC in the individuals with persistent vitamin D deficiency was 1.45-fold (95% *CI*: 1.03, 2.05) higher than those without persistent vitamin D deficiency. The associations between persistent vitamin D deficiency and persistent cardiometabolic risk factors are given in Table 4, and persistent vitamin D deficiency was only significantly associated with persistently high TG [*OR* (95% *CI*): 2.73 (1.71, 4.36)].

**TABLE 3:** The joint effect [*RR* (95% *CI*)]^a^ of persistent vitamin D deficiency and persistent obesity on the cardiometabolic risk factors incident.

Cardiometabolic risk factors	Persistent vitamin D deficiency	Persistent obesity	Case	*N*	*CIR* (%)	*RR* (95% *CI*)	*P*_*interaction*_ ^b^
**Hypertension**							0.391
	–	–	361	5,181	6.9 (6.3∼7.7)	1.00 (Ref)	
	+	–	56	1,004	5.6 (4.2∼7.2)	0.85 (0.63∼1.13)	
	–	+	465	2,306	20.2 (18.5∼21.9)	1.52 (1.25∼1.87)***	
	+	+	73	366	20.0 (16.0∼24.4)	1.52 (1.12∼2.06)**	
**Hyperglycemia**							0.189
	–	–	393	5,194	7.6 (6.9∼8.3)	1.00 (Ref)	
	+	–	67	967	6.9 (5.4∼8.7)	1.01 (0.77∼1.32)	
	–	+	284	2,775	10.2 (9.1∼11.4)	1.18 (0.93∼1.48)	
	+	+	52	416	12.5 (9.5∼16.1)	1.54 (1.09∼2.19)*	
**High TC**							0.963
	–	–	138	5,558	2.5 (2.1∼2.9)	1.00 (Ref)	
	+	–	47	1,063	4.4 (3.3∼5.8)	1.45 (1.03∼2.05)*	
	–	+	97	3,123	3.1 (2.5∼3.8)	1.82 (1.24∼2.69)**	
	+	+	29	467	6.2 (4.2∼8.8)	2.62 (1.57∼4.35)***	
**High LDL-C**							0.762
	–	–	76	5,582	1.4 (1.1∼1.7)	1.00 (Ref)	
	+	–	16	1,068	1.5 (0.8∼2.4)	1.58 (0.91∼2.76)	
	–	+	138	3,088	4.5 (3.8∼5.3)	1.94 (1.31∼2.87)***	
	+	+	22	464	4.7 (3.0∼7.1)	2.75 (1.56∼4.83)***	
**Low HDL-C**							0.551
	–	–	123	5,204	2.4 (2.0∼2.8)	1.00 (Ref)	
	+	–	16	1,009	1.6 (0.9∼2.6)	0.59 (0.35∼1.01)	
	–	+	148	2,399	6.2 (5.2∼7.2)	1.59 (1.12∼2.25)*	
	+	+	21	367	5.7 (3.6∼8.6)	1.16 (0.67∼2.04)	
**High TG**							0.594
	–	–	56	5,641	1.0 (0.8∼1.3)	1.00 (Ref)	
	+	–	11	1,076	1.0 (0.5∼1.8)	1.40 (0.73∼2.72)	
	–	+	154	2,918	5.3 (4.5∼6.2)	3.16 (2.11∼4.72)***	
	+	+	32	426	7.5 (5.2∼10.4)	5.43 (3.21∼9.20)***	

*CIR, cumulative incidence rate; RR, risk ratio; CI, confidence interval; TC, total cholesterol; LDL-C, low-density lipoprotein cholesterol; HDL-C, high-density lipoprotein cholesterol; TG, triglycerides.*

*^a^Model was adjusted for age, sex, smoking, drinking, physical activity, dietary habits, BMI, family history of cardiometabolic risk factors, vitamin D supplements, and sexual maturity.*

*^b^P_interaction_ represents significance of product term of persistent vitamin D deficiency and persistent obesity in the log-binomial model.*

*0.01 ≤ P < 0.05; **0.001 ≤ P < 0.01; ***P < 0.001.

**TABLE 4:** Associations between persistent vitamin D deficiency and persistent cardiometabolic risk factors [*OR* (95% *CI*)]^a^.

Persistent cardiometabolic risk factors	Persistent vitamin D deficiency	Case	*OR* (95% *CI*)	*P-*value
**Hypertension**				
	–	515	1.00 (Ref)	
	+	76	0.96 (0.73∼1.27)	0.789
**Hyperglycemia**				
	–	298	1.00 (Ref)	
	+	70	1.30 (0.98∼1.74)	0.070
**High TC**				
	–	108	1.00 (Ref)	
	+	25	1.01 (0.63∼1.62)	0.958
**High LDL-C**				
	–	97	1.00 (Ref)	
	+	24	1.45 (0.89∼2.36)	0.135
**Low HDL-C**				
	–	386	1.00 (Ref)	
	+	69	1.13 (0.84∼1.52)	0.404
**High TG**				
	–	100	1.00 (Ref)	
	+	33	2.73 (1.71∼4.36)	<0.001

*OR, odds ratio; CI, confidence interval; TC, total cholesterol; LDL-C, low-density lipoprotein cholesterol; HDL-C, high-density lipoprotein cholesterol; TG, triglycerides.*

*^a^Model was adjusted for age, sex, smoking, drinking, physical activity, dietary habits, BMI, family history of cardiometabolic risk factors, vitamin D supplements, and sexual maturity.*

## Discussion

Vitamin D deficiency commonly coexists with cardiometabolic risk factors. However, the association between them remains highly contentious. So far as we know, this is the first population-based cohort study to explore the impacts of the longitudinal vitamin D trajectory on cardiometabolic risk factors in children. We found that children with persistent vitamin D deficiency had significantly increased risks of high TC, LDL-C, and TG. Moreover, vitamin D deficiency at baseline and that at follow-up were independently associated with high TC and TG, respectively. Our findings indicate that the persistence of vitamin D deficiency may increase the risk of dyslipidemia in children, and vitamin D could have short- and long-term effects on TG and TC metabolism, respectively.

Despite copious observational studies suggesting the potential role of vitamin D deficiency as a cardiovascular disease indicator (7–9, 19, 20), there is a lack of reliable evidence derived from prospective studies. Different from a recent birth cohort study reporting the association of low vitamin D status and trajectory in early life with the increased risk of elevated SBP during childhood and adolescence (21), our results failed to show any effects of persistent vitamin D deficiency on the risk of hypertension after the adjustment of BMI. The associations they found were independent of maternal cardiometabolic condition, but attenuated when further adjusted for the children’s current overweight and obesity, which indicated they were partly mediated by the children’s weight status. Besides weight status, skin nitric oxide (NO) caused by solar radiation via ultraviolet-B might induce the associations between them too (22). Thus, the beneficial effects on blood pressure may result from UV radiation other than vitamin D production.

In previous cross-sectional studies, vitamin D deficiency was reported to be associated with the increased risk of hyperglycemia in both United States and Chinese children (6, 7). However, we failed to observe any association of vitamin D trajectory with hyperglycemia using a prospective analysis in the current study. Using the Mendelian randomization analysis, Zheng et al. did not find a causal association between 25(OH)D and type 2 diabetes in European either (23). A clinical trial including 33,951 postmenopausal women found that 400 IU of vitamin D supplementation did not reduce the risk of diabetes during a 7-year follow-up (24). Thus, further research is needed to clarify the effect of vitamin D on glycemic control.

The inverse associations between vitamin D and atherogenic lipid profiles have been reported in previous epidemiological studies (7, 25). Our prospective study extended prior findings and was the first to explore the effect of longitudinal vitamin D trajectory on dyslipidemia in childhood. Mainly, we found that vitamin D trajectories were related to dyslipidemia rather than other cardiometabolic risk factors: persistent vitamin D deficiency was associated with the increased risk of developing high TC, LDL-C, and TG. The Women’s Health Initiative Investigators found that daily intake of 400 IU of vitamin D plus 1000 mg of calcium was correlated with a 4.5 mg/dl (*P* = 0.03) decrease in LDL-C, and higher vitamin D levels were related to lower TG levels (*P* < 0.001) (26). Another Mendelian randomization reported that vitamin D related single nucleotide polymorphisms can result in genetically increased remnant lipoprotein-cholesterol (27). Interestingly, we found that the low serum 25(OH)D concentrations at baseline and follow-up appeared to play an important role in the development of high TC and TG, respectively. These results indicated that vitamin D deficiency might have long-term effects on TC, as well as short-term effects on TG. A cross-sectional analysis of 243 non-obese healthy children aged 9–18 years found the vitamin D deficient group had a significantly higher level of TG (25). In our subgroup analyses of children who were non-obese at both baseline and follow-up, persistent vitamin D deficiency was only associated with high TC. Although the biological mechanism of vitamin D affecting lipid metabolism remains unclear, one hypothesis has been established that vitamin D might decrease the formation or secretion of hepatic triglyceride by promoting calcium absorption (4). This hypothesis was also supported by the prior studies which reported the associations between inadequate calcium intake and more atherogenic lipid metabolism (28). Vitamin D, a fat-soluble sterol distributed in various tissues of the body, may decrease its bioavailability due to excess storage in the body fat compartment (29). Hence, previous cross-sectional studies explored the joint effects of vitamin D deficiency and obesity on cardiovascular diseases, and found they had synergistic effects on hyperglycemia or diabetes (7, 30). However, our current cohort study did not find any modification of persistent obesity on the associations between persistent vitamin D deficiency and cardiovascular risk factors.

One of the unique features of this study was the large sample of population-based children cohort. We utilized the 2-time points measurements of 25(OH)D concentrations to classify possible vitamin D trajectories in children, and estimated the cumulative incidence rates using new-onset cardiometabolic risk factors during follow-up. The reverse causality could be avoided in our research by using a prospective design. Nevertheless, the present study had a few limitations. First, our study population was dominantly Chinese children, and therefore, the generalizability may be limited due to a lack of other ethnic groups. Second, the information of dietary behaviors and physical activity was collected using a questionnaire and thus was potentially susceptible to reporting bias. Although our study used an observational design, we believe that our results have a large public health significance. This statistical evidence supports the notion that the burden of dyslipidemia in children may be reduced by maintaining an adequate level of serum 25(OH)D concentrations. Thus, vitamin D supplements may be a less expensive and more practical means of reducing the burden of childhood dyslipidemia compared to lipid-lowering drugs.

In summary, by analyzing the data from a large population-based cohort, we identified the causal associations between persistent vitamin D deficiency status and the risks of developing high TC, LDL-C, and TG in childhood. Additionally, low vitamin D status is suggested to have long-term effects on TC and short-term effects on TG. Our findings raise the possibility that maintenance of sufficient 25(OH)D concentrations during childhood may prevent children from dyslipidemia.

## Data Availability Statement

The raw data supporting the conclusions of this article will be made available by the authors, without undue reservation.

## Ethics Statement

The studies involving human participants were reviewed and approved by the Institutional Review Boards of Capital Institute of Pediatrics. Written informed consent to participate in this study was provided by the participants’ legal guardian/next of kin.

## Author Contributions

JM: conceptualization, methodology, supervision, and writing—riginal draft. PX: data curation, formal analysis, and writing—original draft preparation. HC: investigation, writing, reviewing, and editing. HL: visualization and software. DH and XZ: investigation. XX: methodology, supervision, and investigation. All authors contributed to the article and approved the submitted version.

## Conflict of Interest

The authors declare that the research was conducted in the absence of any commercial or financial relationships that could be construed as a potential conflict of interest.

## Publisher’s Note

All claims expressed in this article are solely those of the authors and do not necessarily represent those of their affiliated organizations, or those of the publisher, the editors and the reviewers. Any product that may be evaluated in this article, or claim that may be made by its manufacturer, is not guaranteed or endorsed by the publisher.

## Footnotes

1
www.cran.r-project.org
