# Supplementary material for: Vitamin D Trajectories and Cardiometabolic Risk Factors During Childhood: A Large Population-Based Prospective Cohort Study
Source: Front Cardiovasc Med. 2022 Mar 16;9:836376. doi: 10.3389/fcvm.2022.836376 (PMC8966377; doi:10.3389/fcvm.2022.836376)
Supplement: Supplementary file 1 [file Table_1.DOCX]

Supplementary Material

# Supplementary Table 1

Age- and height-specific blood pressure percentiles for Chinese boys aged 3-17 years

| Age, y | Height, cm | SBP, mm Hg | | | |  | DBP, mm Hg | | | |
| --- | --- | --- | --- | --- | --- | --- | --- | --- | --- | --- |
|  |  | P_50_ | P_90_ | P_95_ | P_99_ |  | P_50_ | P_90_ | P_95_ | P_99_ |
| 3 | <96 | 88 | 99 | 102 | 108 |  | 54 | 62 | 65 | 72 |
|  | 96~97 | 88 | 100 | 103 | 109 |  | 54 | 63 | 65 | 72 |
|  | 98~100 | 89 | 101 | 104 | 110 |  | 54 | 63 | 66 | 72 |
|  | 101~103 | 90 | 102 | 105 | 112 |  | 54 | 63 | 66 | 73 |
|  | 104~106 | 91 | 103 | 107 | 113 |  | 55 | 63 | 66 | 73 |
|  | 107~108 | 92 | 104 | 107 | 114 |  | 55 | 63 | 66 | 73 |
|  | ≥109 | 93 | 105 | 108 | 115 |  | 55 | 63 | 66 | 73 |
| 4 | <102 | 89 | 101 | 104 | 111 |  | 55 | 64 | 67 | 74 |
|  | 102~104 | 90 | 102 | 105 | 111 |  | 55 | 64 | 67 | 74 |
|  | 105~107 | 91 | 103 | 106 | 113 |  | 55 | 64 | 67 | 74 |
|  | 108~110 | 92 | 104 | 108 | 114 |  | 56 | 64 | 67 | 74 |
|  | 111~113 | 93 | 106 | 109 | 115 |  | 56 | 64 | 67 | 74 |
|  | 114~116 | 94 | 107 | 110 | 117 |  | 56 | 65 | 68 | 75 |
|  | ≥117 | 95 | 107 | 111 | 117 |  | 56 | 65 | 68 | 75 |
| 5 | <109 | 92 | 104 | 107 | 114 |  | 56 | 65 | 68 | 75 |
|  | 109~110 | 92 | 104 | 107 | 114 |  | 56 | 65 | 68 | 75 |
|  | 111~113 | 93 | 105 | 109 | 115 |  | 56 | 65 | 68 | 75 |
|  | 114~117 | 94 | 106 | 110 | 117 |  | 57 | 65 | 69 | 76 |
|  | 118~120 | 95 | 108 | 111 | 118 |  | 57 | 66 | 69 | 76 |
|  | 121~123 | 96 | 109 | 112 | 119 |  | 58 | 67 | 70 | 77 |
|  | ≥124 | 97 | 110 | 113 | 120 |  | 58 | 67 | 70 | 77 |
| 6 | <114 | 93 | 105 | 109 | 115 |  | 57 | 66 | 69 | 76 |
|  | 114~116 | 94 | 106 | 110 | 116 |  | 57 | 66 | 69 | 76 |
|  | 117~119 | 95 | 107 | 111 | 117 |  | 58 | 66 | 69 | 77 |
|  | 120~123 | 96 | 108 | 112 | 119 |  | 58 | 67 | 70 | 78 |
|  | 124~126 | 97 | 110 | 113 | 120 |  | 59 | 68 | 71 | 78 |
|  | 127~129 | 98 | 111 | 115 | 121 |  | 59 | 69 | 72 | 79 |
|  | ≥130 | 99 | 112 | 116 | 123 |  | 60 | 69 | 73 | 80 |
| 7 | <118 | 94 | 106 | 110 | 117 |  | 58 | 67 | 70 | 77 |
|  | 118~120 | 95 | 107 | 111 | 118 |  | 58 | 67 | 70 | 78 |
|  | 121~123 | 96 | 108 | 112 | 119 |  | 59 | 68 | 71 | 78 |
|  | 124~127 | 97 | 110 | 113 | 120 |  | 59 | 68 | 72 | 79 |
|  | 128~131 | 98 | 112 | 115 | 122 |  | 60 | 70 | 73 | 81 |
|  | 132~135 | 100 | 113 | 117 | 124 |  | 61 | 71 | 74 | 82 |
|  | ≥136 | 100 | 114 | 117 | 125 |  | 62 | 71 | 74 | 82 |
| 8 | <121 | 95 | 108 | 111 | 118 |  | 59 | 68 | 71 | 78 |
|  | 121~123 | 95 | 108 | 112 | 119 |  | 59 | 68 | 71 | 79 |
|  | 124~127 | 97 | 110 | 113 | 120 |  | 60 | 69 | 72 | 80 |
|  | 128~132 | 98 | 111 | 115 | 122 |  | 61 | 70 | 73 | 81 |
|  | 133~136 | 99 | 113 | 117 | 124 |  | 62 | 71 | 74 | 82 |
|  | 137~139 | 101 | 114 | 118 | 125 |  | 62 | 72 | 75 | 83 |
|  | ≥140 | 102 | 115 | 119 | 127 |  | 63 | 73 | 76 | 84 |
| 9 | <125 | 96 | 109 | 112 | 119 |  | 60 | 69 | 72 | 80 |
|  | 125~128 | 96 | 109 | 113 | 120 |  | 60 | 69 | 73 | 80 |
|  | 129~132 | 98 | 111 | 115 | 122 |  | 61 | 71 | 74 | 82 |
|  | 133~137 | 99 | 113 | 117 | 124 |  | 62 | 72 | 75 | 83 |
|  | 138~142 | 101 | 115 | 119 | 126 |  | 63 | 73 | 76 | 84 |
|  | 143~145 | 102 | 116 | 120 | 128 |  | 64 | 73 | 77 | 85 |
|  | ≥146 | 103 | 117 | 121 | 129 |  | 64 | 74 | 77 | 85 |
| 10 | <130 | 97 | 110 | 114 | 121 |  | 61 | 70 | 74 | 81 |
|  | 130~132 | 98 | 111 | 115 | 122 |  | 62 | 71 | 74 | 82 |
|  | 133~137 | 99 | 113 | 116 | 124 |  | 62 | 72 | 75 | 83 |
|  | 138~142 | 101 | 115 | 119 | 126 |  | 63 | 73 | 77 | 85 |
|  | 143~147 | 102 | 117 | 120 | 128 |  | 64 | 74 | 77 | 85 |
|  | 148~151 | 104 | 118 | 122 | 130 |  | 64 | 74 | 77 | 86 |
|  | ≥152 | 105 | 119 | 123 | 131 |  | 64 | 74 | 77 | 86 |
| 11 | <134 | 98 | 111 | 115 | 122 |  | 62 | 72 | 75 | 83 |
|  | 134~137 | 99 | 112 | 116 | 124 |  | 63 | 72 | 76 | 84 |
|  | 138~142 | 100 | 114 | 118 | 126 |  | 64 | 73 | 77 | 85 |
|  | 143~148 | 102 | 116 | 120 | 128 |  | 64 | 74 | 78 | 86 |
|  | 149~153 | 104 | 119 | 123 | 130 |  | 64 | 74 | 78 | 86 |
|  | 154~157 | 106 | 120 | 124 | 132 |  | 64 | 74 | 78 | 86 |
|  | ≥158 | 106 | 121 | 125 | 133 |  | 64 | 74 | 78 | 86 |
| 12 | <140 | 100 | 113 | 117 | 125 |  | 64 | 73 | 77 | 85 |
|  | 140~144 | 101 | 115 | 119 | 126 |  | 64 | 74 | 78 | 86 |
|  | 145~149 | 102 | 117 | 121 | 128 |  | 65 | 75 | 78 | 86 |
|  | 150~155 | 104 | 119 | 123 | 131 |  | 65 | 75 | 78 | 86 |
|  | 156~160 | 106 | 121 | 125 | 133 |  | 65 | 75 | 78 | 86 |
|  | 161~164 | 108 | 123 | 127 | 135 |  | 65 | 75 | 78 | 87 |
|  | ≥165 | 108 | 124 | 128 | 136 |  | 65 | 75 | 78 | 87 |
| 13 | <147 | 102 | 116 | 120 | 128 |  | 65 | 75 | 78 | 86 |
|  | 147~151 | 103 | 117 | 121 | 129 |  | 65 | 75 | 78 | 87 |
|  | 152~156 | 104 | 119 | 123 | 131 |  | 65 | 75 | 79 | 87 |
|  | 157~162 | 106 | 121 | 125 | 133 |  | 65 | 75 | 79 | 87 |
|  | 163~167 | 108 | 123 | 128 | 136 |  | 65 | 75 | 79 | 87 |
|  | 168~171 | 110 | 125 | 130 | 138 |  | 66 | 76 | 79 | 87 |
|  | ≥172 | 110 | 126 | 130 | 139 |  | 66 | 76 | 79 | 88 |
| 14 | <154 | 103 | 118 | 122 | 130 |  | 65 | 75 | 79 | 87 |
|  | 154~157 | 104 | 119 | 124 | 132 |  | 65 | 75 | 79 | 87 |
|  | 158~162 | 106 | 121 | 125 | 133 |  | 65 | 75 | 79 | 87 |
|  | 163~167 | 108 | 123 | 128 | 136 |  | 65 | 75 | 79 | 87 |
|  | 168~172 | 109 | 125 | 130 | 138 |  | 66 | 76 | 79 | 88 |
|  | 173~176 | 111 | 127 | 131 | 140 |  | 66 | 76 | 80 | 88 |
|  | ≥177 | 112 | 128 | 133 | 141 |  | 67 | 77 | 80 | 89 |
| 15 | <158 | 105 | 120 | 124 | 132 |  | 65 | 76 | 79 | 87 |
|  | 158~161 | 106 | 121 | 125 | 133 |  | 65 | 76 | 79 | 87 |
|  | 162~166 | 107 | 122 | 127 | 135 |  | 66 | 76 | 79 | 88 |
|  | 167~170 | 109 | 124 | 128 | 137 |  | 66 | 76 | 80 | 88 |
|  | 171~174 | 110 | 126 | 131 | 139 |  | 66 | 77 | 80 | 89 |
|  | 175~178 | 112 | 128 | 132 | 141 |  | 67 | 77 | 81 | 89 |
|  | ≥179 | 113 | 129 | 133 | 142 |  | 67 | 77 | 81 | 90 |
| 16 | <161 | 105 | 121 | 125 | 133 |  | 66 | 76 | 79 | 88 |
|  | 161~164 | 106 | 121 | 126 | 134 |  | 66 | 76 | 79 | 88 |
|  | 165~168 | 107 | 123 | 127 | 136 |  | 66 | 76 | 80 | 88 |
|  | 169~172 | 109 | 125 | 129 | 138 |  | 66 | 76 | 80 | 88 |
|  | 173~176 | 111 | 126 | 131 | 140 |  | 67 | 77 | 80 | 89 |
|  | 177~179 | 112 | 128 | 133 | 141 |  | 67 | 77 | 81 | 90 |
|  | ≥180 | 113 | 129 | 134 | 142 |  | 67 | 78 | 81 | 90 |
| 17 | <163 | 106 | 121 | 126 | 134 |  | 66 | 76 | 80 | 88 |
|  | 163~165 | 107 | 122 | 126 | 135 |  | 66 | 76 | 80 | 88 |
|  | 166~169 | 108 | 124 | 128 | 136 |  | 66 | 76 | 80 | 88 |
|  | 170~173 | 109 | 125 | 130 | 138 |  | 67 | 77 | 80 | 89 |
|  | 174~177 | 111 | 127 | 131 | 140 |  | 67 | 77 | 81 | 89 |
|  | 178~180 | 112 | 129 | 133 | 142 |  | 67 | 78 | 81 | 90 |
|  | ≥181 | 113 | 129 | 134 | 143 |  | 68 | 78 | 82 | 90 |

# Supplementary Table 2

Age- and height-specific blood pressure percentiles for Chinese girls aged 3-17 years

| Age, y | Height, cm | SBP, mm Hg | | | |  | DBP, mm Hg | | | |
| --- | --- | --- | --- | --- | --- | --- | --- | --- | --- | --- |
|  |  | P_50_ | P_90_ | P_95_ | P_99_ |  | P_50_ | P_90_ | P_95_ | P_99_ |
| 3 | <95 | 87 | 99 | 102 | 108 |  | 55 | 63 | 67 | 74 |
|  | 95~96 | 88 | 99 | 103 | 109 |  | 55 | 63 | 67 | 74 |
|  | 97~99 | 88 | 100 | 103 | 110 |  | 55 | 64 | 67 | 74 |
|  | 100~102 | 89 | 101 | 104 | 111 |  | 55 | 64 | 67 | 74 |
|  | 103~105 | 90 | 102 | 105 | 112 |  | 55 | 64 | 67 | 74 |
|  | 106~107 | 91 | 103 | 106 | 113 |  | 55 | 64 | 67 | 75 |
|  | ≥108 | 91 | 103 | 107 | 113 |  | 56 | 64 | 67 | 75 |
| 4 | <101 | 89 | 101 | 105 | 111 |  | 56 | 64 | 67 | 75 |
|  | 101~103 | 89 | 101 | 105 | 111 |  | 56 | 64 | 67 | 75 |
|  | 104~106 | 90 | 102 | 106 | 112 |  | 56 | 64 | 67 | 75 |
|  | 107~109 | 91 | 103 | 107 | 113 |  | 56 | 64 | 67 | 75 |
|  | 110~112 | 92 | 104 | 107 | 114 |  | 56 | 65 | 68 | 75 |
|  | 113~114 | 93 | 105 | 109 | 115 |  | 56 | 65 | 68 | 76 |
|  | ≥115 | 93 | 105 | 109 | 115 |  | 56 | 65 | 68 | 76 |
| 5 | <108 | 91 | 103 | 106 | 113 |  | 56 | 65 | 68 | 76 |
|  | 108~109 | 91 | 103 | 107 | 113 |  | 56 | 65 | 68 | 76 |
|  | 110~112 | 92 | 104 | 107 | 114 |  | 56 | 65 | 68 | 76 |
|  | 113~116 | 93 | 105 | 109 | 115 |  | 57 | 65 | 68 | 76 |
|  | 117~119 | 93 | 106 | 109 | 116 |  | 57 | 66 | 69 | 77 |
|  | 120~122 | 94 | 107 | 111 | 117 |  | 58 | 66 | 70 | 77 |
|  | ≥123 | 95 | 108 | 111 | 118 |  | 58 | 67 | 70 | 78 |
| 6 | <113 | 92 | 104 | 108 | 115 |  | 57 | 65 | 69 | 76 |
|  | 113~114 | 92 | 105 | 108 | 115 |  | 57 | 66 | 69 | 77 |
|  | 115~118 | 93 | 106 | 109 | 116 |  | 57 | 66 | 69 | 77 |
|  | 119~121 | 94 | 107 | 110 | 117 |  | 58 | 67 | 70 | 78 |
|  | 122~125 | 95 | 108 | 112 | 118 |  | 58 | 67 | 71 | 79 |
|  | 126~128 | 96 | 109 | 113 | 119 |  | 59 | 68 | 71 | 79 |
|  | ≥129 | 97 | 110 | 114 | 121 |  | 59 | 69 | 72 | 80 |
| 7 | <116 | 93 | 105 | 109 | 115 |  | 57 | 66 | 69 | 77 |
|  | 116~118 | 93 | 106 | 109 | 116 |  | 57 | 66 | 69 | 77 |
|  | 119~122 | 94 | 107 | 110 | 117 |  | 58 | 67 | 70 | 78 |
|  | 123~126 | 95 | 108 | 112 | 119 |  | 59 | 68 | 71 | 79 |
|  | 127~130 | 96 | 109 | 113 | 120 |  | 59 | 69 | 72 | 80 |
|  | 131~133 | 97 | 111 | 114 | 122 |  | 60 | 69 | 73 | 81 |
|  | ≥134 | 98 | 112 | 115 | 122 |  | 61 | 70 | 73 | 82 |
| 8 | <120 | 94 | 106 | 110 | 116 |  | 58 | 67 | 70 | 78 |
|  | 120~122 | 94 | 107 | 111 | 117 |  | 58 | 67 | 71 | 79 |
|  | 123~126 | 95 | 108 | 112 | 119 |  | 59 | 68 | 71 | 79 |
|  | 127~131 | 96 | 109 | 113 | 120 |  | 60 | 69 | 72 | 80 |
|  | 132~135 | 98 | 111 | 115 | 122 |  | 61 | 70 | 73 | 82 |
|  | 136~138 | 99 | 112 | 116 | 123 |  | 61 | 71 | 74 | 83 |
|  | ≥139 | 100 | 113 | 117 | 124 |  | 62 | 71 | 75 | 83 |
| 9 | <124 | 95 | 108 | 111 | 118 |  | 59 | 68 | 71 | 79 |
|  | 124~127 | 95 | 108 | 112 | 119 |  | 59 | 68 | 72 | 80 |
|  | 128~132 | 97 | 110 | 113 | 120 |  | 60 | 69 | 73 | 81 |
|  | 133~136 | 98 | 111 | 115 | 122 |  | 61 | 71 | 74 | 82 |
|  | 137~141 | 100 | 113 | 117 | 124 |  | 62 | 72 | 75 | 84 |
|  | 142~145 | 101 | 114 | 118 | 125 |  | 63 | 72 | 76 | 84 |
|  | ≥146 | 102 | 115 | 119 | 126 |  | 63 | 73 | 76 | 85 |
| 10 | <130 | 96 | 109 | 113 | 120 |  | 60 | 69 | 73 | 81 |
|  | 130~133 | 97 | 110 | 114 | 121 |  | 61 | 70 | 73 | 82 |
|  | 134~138 | 99 | 112 | 116 | 123 |  | 62 | 71 | 75 | 83 |
|  | 139~143 | 100 | 113 | 117 | 124 |  | 63 | 72 | 76 | 84 |
|  | 144~147 | 101 | 115 | 119 | 126 |  | 63 | 73 | 76 | 85 |
|  | 148~151 | 103 | 116 | 120 | 128 |  | 63 | 73 | 77 | 85 |
|  | ≥152 | 103 | 117 | 121 | 129 |  | 64 | 73 | 77 | 86 |
| 11 | <136 | 98 | 112 | 115 | 122 |  | 62 | 71 | 75 | 83 |
|  | 136~139 | 99 | 113 | 116 | 123 |  | 62 | 72 | 75 | 84 |
|  | 140~144 | 101 | 114 | 118 | 125 |  | 63 | 73 | 76 | 85 |
|  | 145~149 | 102 | 116 | 120 | 127 |  | 64 | 73 | 77 | 86 |
|  | 150~154 | 103 | 117 | 121 | 128 |  | 64 | 74 | 77 | 86 |
|  | 155~157 | 104 | 118 | 122 | 129 |  | 64 | 74 | 77 | 86 |
|  | ≥158 | 104 | 118 | 122 | 130 |  | 64 | 74 | 77 | 86 |
| 12 | <142 | 100 | 113 | 117 | 124 |  | 63 | 73 | 76 | 85 |
|  | 142~145 | 101 | 114 | 118 | 125 |  | 63 | 73 | 77 | 85 |
|  | 146~150 | 102 | 116 | 120 | 127 |  | 64 | 74 | 77 | 86 |
|  | 151~154 | 103 | 117 | 121 | 129 |  | 64 | 74 | 78 | 86 |
|  | 155~158 | 104 | 118 | 122 | 130 |  | 64 | 74 | 78 | 87 |
|  | 159~162 | 105 | 119 | 123 | 130 |  | 64 | 74 | 78 | 87 |
|  | ≥163 | 105 | 119 | 123 | 131 |  | 64 | 74 | 78 | 87 |
| 13 | <147 | 101 | 115 | 119 | 126 |  | 64 | 74 | 77 | 86 |
|  | 147~149 | 102 | 116 | 120 | 127 |  | 64 | 74 | 78 | 87 |
|  | 150~153 | 103 | 117 | 121 | 128 |  | 64 | 74 | 78 | 87 |
|  | 154~157 | 104 | 118 | 122 | 129 |  | 65 | 74 | 78 | 87 |
|  | 158~161 | 105 | 119 | 123 | 130 |  | 65 | 74 | 78 | 87 |
|  | 162~164 | 105 | 119 | 123 | 131 |  | 65 | 74 | 78 | 87 |
|  | ≥165 | 105 | 119 | 123 | 131 |  | 65 | 75 | 78 | 87 |
| 14 | <149 | 102 | 116 | 120 | 127 |  | 65 | 74 | 78 | 87 |
|  | 149~152 | 103 | 117 | 121 | 128 |  | 65 | 75 | 78 | 87 |
|  | 153~155 | 104 | 118 | 122 | 129 |  | 65 | 75 | 78 | 87 |
|  | 156~159 | 104 | 118 | 122 | 130 |  | 65 | 75 | 78 | 87 |
|  | 160~163 | 105 | 119 | 123 | 130 |  | 65 | 75 | 78 | 87 |
|  | 164~166 | 105 | 119 | 123 | 131 |  | 65 | 75 | 79 | 87 |
|  | ≥167 | 106 | 120 | 124 | 131 |  | 65 | 75 | 79 | 88 |
| 15 | <151 | 103 | 116 | 120 | 128 |  | 65 | 75 | 79 | 87 |
|  | 151~152 | 103 | 117 | 121 | 128 |  | 65 | 75 | 79 | 88 |
|  | 153~156 | 104 | 118 | 122 | 129 |  | 65 | 75 | 79 | 88 |
|  | 157~160 | 105 | 119 | 123 | 130 |  | 65 | 75 | 79 | 88 |
|  | 161~163 | 105 | 119 | 123 | 131 |  | 65 | 75 | 79 | 88 |
|  | 164~166 | 105 | 120 | 124 | 131 |  | 65 | 75 | 79 | 88 |
|  | ≥167 | 106 | 120 | 124 | 131 |  | 65 | 75 | 79 | 88 |
| 16 | <151 | 103 | 117 | 121 | 128 |  | 65 | 75 | 79 | 88 |
|  | 151~153 | 103 | 117 | 121 | 129 |  | 65 | 75 | 79 | 88 |
|  | 154~157 | 104 | 118 | 122 | 130 |  | 65 | 75 | 79 | 88 |
|  | 158~160 | 105 | 119 | 123 | 130 |  | 65 | 75 | 79 | 88 |
|  | 161~164 | 105 | 119 | 123 | 131 |  | 66 | 76 | 79 | 88 |
|  | 165~167 | 106 | 120 | 124 | 131 |  | 66 | 76 | 79 | 88 |
|  | ≥168 | 106 | 120 | 124 | 132 |  | 66 | 76 | 79 | 88 |
| 17 | <152 | 103 | 117 | 121 | 129 |  | 66 | 76 | 79 | 88 |
|  | 152~154 | 104 | 118 | 122 | 129 |  | 66 | 76 | 79 | 89 |
|  | 155~157 | 104 | 118 | 122 | 130 |  | 66 | 76 | 80 | 89 |
|  | 158~161 | 105 | 119 | 123 | 130 |  | 66 | 76 | 80 | 89 |
|  | 162~164 | 105 | 119 | 124 | 131 |  | 66 | 76 | 80 | 89 |
|  | 165~167 | 106 | 120 | 124 | 132 |  | 66 | 76 | 80 | 89 |
|  | ≥168 | 106 | 120 | 124 | 132 |  | 66 | 76 | 80 | 89 |

# Supplementary Table 3

Gender- and age-specific abnormal lipid cutpoints and corresponding centiles for Chinese children aged 6-18 years

| **Age, y** | **TC, mmol/L** | | | | **LDL-C, mmol/L** | | | | **HDL-C, mmol/L** | | **TG, mmol/L** | | | |
| --- | --- | --- | --- | --- | --- | --- | --- | --- | --- | --- | --- | --- | --- | --- |
|  | **Borderline-High** | | **High** | | **Borderline-High** | | **High** | | **Low** | | **Borderline-High** | | **High** | |
|  | **Boys**  **(98.0^th^)** | **Girls**  **(96.6^th^)** | **Boys**  **(99.8^th^)** | **Girls**  **(99.6^th^)** | **Boys**  **(97.4^th^)** | **Girls**  **(97.3^th^)** | **Boys**  **(99.5^th^)** | **Girls**  **(99.4^th^)** | **Boys**  **(12.4^th^)** | **Girls**  **(4.7^th^)** | **Boys**  **(93.2^th^)** | **Girls**  **(96.9^th^)** | **Boys**  **(97.7^th^)** | **Girls**  **(99.3^th^)** |
| 6 | 5.85 | 5.59 | 6.98 | 6.67 | 3.62 | 3.71 | 4.37 | 4.47 | 1.15 | 1.02 | 1.44 | 1.97 | 1.96 | 2.94 |
| 7 | 5.78 | 5.55 | 6.89 | 6.62 | 3.56 | 3.67 | 4.30 | 4.41 | 1.15 | 1.01 | 1.40 | 1.92 | 1.90 | 2.83 |
| 8 | 5.72 | 5.49 | 6.82 | 6.56 | 3.53 | 3.61 | 4.25 | 4.34 | 1.15 | 1.01 | 1.40 | 1.90 | 1.89 | 2.78 |
| 9 | 5.70 | 5.42 | 6.79 | 6.47 | 3.52 | 3.53 | 4.24 | 4.25 | 1.15 | 1.00 | 1.44 | 1.91 | 1.95 | 2.77 |
| 10 | 5.66 | 5.34 | 6.75 | 6.37 | 3.50 | 3.46 | 4.22 | 4.17 | 1.13 | 0.99 | 1.50 | 1.95 | 2.02 | 2.80 |
| 11 | 5.57 | 5.26 | 6.64 | 6.27 | 3.46 | 3.40 | 4.17 | 4.09 | 1.11 | 0.98 | 1.54 | 1.98 | 2.08 | 2.82 |
| 12 | 5.44 | 5.19 | 6.49 | 6.19 | 3.39 | 3.35 | 4.09 | 4.03 | 1.09 | 0.97 | 1.56 | 1.97 | 2.11 | 2.79 |
| 13 | 5.32 | 5.15 | 6.34 | 6.15 | 3.34 | 3.35 | 4.03 | 4.03 | 1.07 | 0.98 | 1.57 | 1.94 | 2.13 | 2.72 |
| 14 | 5.24 | 5.17 | 6.25 | 6.17 | 3.32 | 3.39 | 4.00 | 4.08 | 1.06 | 0.99 | 1.59 | 1.88 | 2.15 | 2.62 |
| 15 | 5.22 | 5.22 | 6.23 | 6.23 | 3.33 | 3.45 | 4.02 | 4.16 | 1.05 | 1.00 | 1.61 | 1.81 | 2.18 | 2.51 |
| 16 | 5.23 | 5.26 | 6.24 | 6.28 | 3.37 | 3.49 | 4.07 | 4.20 | 1.04 | 1.01 | 1.63 | 1.75 | 2.21 | 2.41 |
| 17 | 5.23 | 5.25 | 6.24 | 6.26 | 3.40 | 3.47 | 4.11 | 4.17 | 1.02 | 1.01 | 1.67 | 1.72 | 2.26 | 2.34 |
| 18 | 5.20 | 5.20 | 6.20 | 6.20 | 3.40 | 3.40 | 4.10 | 4.10 | 1.00 | 1.00 | 1.70 | 1.70 | 2.30 | 2.30 |

SI conversion factors: To convert TC, LDL-C, and HDL-C to mg/dL, multiply values by 38.67; To convert TG to mg/dL, multiply values by 88.5.

Borderline-High lipid cutpoints were used to define dyslipidemia in the current study.
